# Risk factor analysis of clinical outcomes of total aortic arch replacement and frozen elephant trunk with aortic balloon occlusion

**DOI:** 10.1186/s13019-021-01643-3

**Published:** 2021-09-08

**Authors:** Luchen Wang, Yunfeng Li, Yaojun Dun, Xiaogang Sun

**Affiliations:** Aortic and Vascular Surgery Center, Fuwai Hospital, National Center for Cardiovascular Diseases, Chinese Academy of Medical Sciences and Peking Union Medical College, No.167 North Lishi Road, Xicheng District, Beijing, 100037 China

**Keywords:** Total aortic arch replacement, Frozen elephant trunk, Hypothermic circulatory arrest, Aortic balloon occlusion, Hepatic transaminase, Blood transfusion

## Abstract

**Background:**

Total aortic arch replacement (TAR) with frozen elephant trunk (FET) requires hypothermic circulatory arrest (HCA) for 20 min, which increases the surgical risk. We invented an aortic balloon occlusion (ABO) technique that requires 5 min of HCA on average to perform TAR with FET and investigated the possible merit of this new method in this study.

**Methods:**

This retrospective study included consecutive patients who underwent TAR and FET (consisting of 130 cases of ABO group and 230 cases of conventional group) in Fuwai Hospital between August 2017 and February 2019. In addition to the postoperative complications, the alterations of blood routine tests, alanine transaminase (ALT) and aspartate transaminase (AST) during in-hospital stay were also recorded.

**Results:**

The 30-day mortality rates were similar between ABO group (4.6%) and conventional group (7.8%, P = 0.241). Multivariate analysis showed ABO reduced postoperative acute kidney injury (23.1% vs. 35.7%, P = 0.013) and hepatic injury (12.3% vs. 27.8%, P = 0.001), and maintained similar cost to patients (25.5 vs. 24.9 kUSD, P = 0.298). We also found that AST was high during intensive care unit (ICU) stay and recovered to normal before discharge, while ALT was not as high as AST in ICU but showed a rising tendency before discharge. The platelet count showed a rising tendency on postoperative day 3 and may exceed the preoperative value before discharge.

**Conclusions:**

The ABO achieved the surgical goal of TAR with FET with an improved recovery process during the in-hospital stay.

**Supplementary Information:**

The online version contains supplementary material available at 10.1186/s13019-021-01643-3.

## Introduction

Total aortic arch replacement (TAR) by using the frozen elephant trunk (FET) technique is the standard procedure to treat aortic disease involving repair of the aortic arch and proximal descending thoracic aorta in our hospital [1–3] as well as in many aortic centers in the world [4–12]. Most recently, an aortic balloon occlusion (ABO) technique was applied to TAR and FET that consistently shortened the lower body circulatory arrest (CA) time and further raised the target nadir temperature setting point for CA [13]. This ABO technique allowed continuous perfusion to abdominal end organs during TAR and FET and eliminated the need for prolonged lower body CA, because it required only 5 min (min) on average to set up the ABO device before the lower body CA was ended and the following procedure (the distal aortic anastomosis in TAR and FET) could last as long as needed. The present study investigated the effects of the ABO technique on the postoperative recovery process, clinical outcome, and blood transfusion of the TAR and FET operation, as well as explored the risk factors for these events. By comparing the strength of the protective effect of the ABO technique statistically against the other risk factors, we aimed not only to discover to what extent the ABO technique has improved the clinical results, but also to explore how the other risk factors could affect different clinical results. The significance of the ABO technique among all factors could guide the usage of this approach for each patient according to the preoperative conditions.

## Methods

### Patient selection

We reviewed the in-hospital record of all these patients, which were the routine parameters from our institution and did not constitute any additional burden for the patients. The cases of the consecutive patients who underwent the TAR and FET operation in Fuwai Hospital between August 2017 and February 2019 were reviewed, including those of 130 patients who received the new TAR and FET by ABO and 230 those of patients who received the conventional TAR and FET. The perioperative details were separately recorded and compared to investigate the possible merit of the ABO technique.

### Surgical approach

**Fig. 1:**
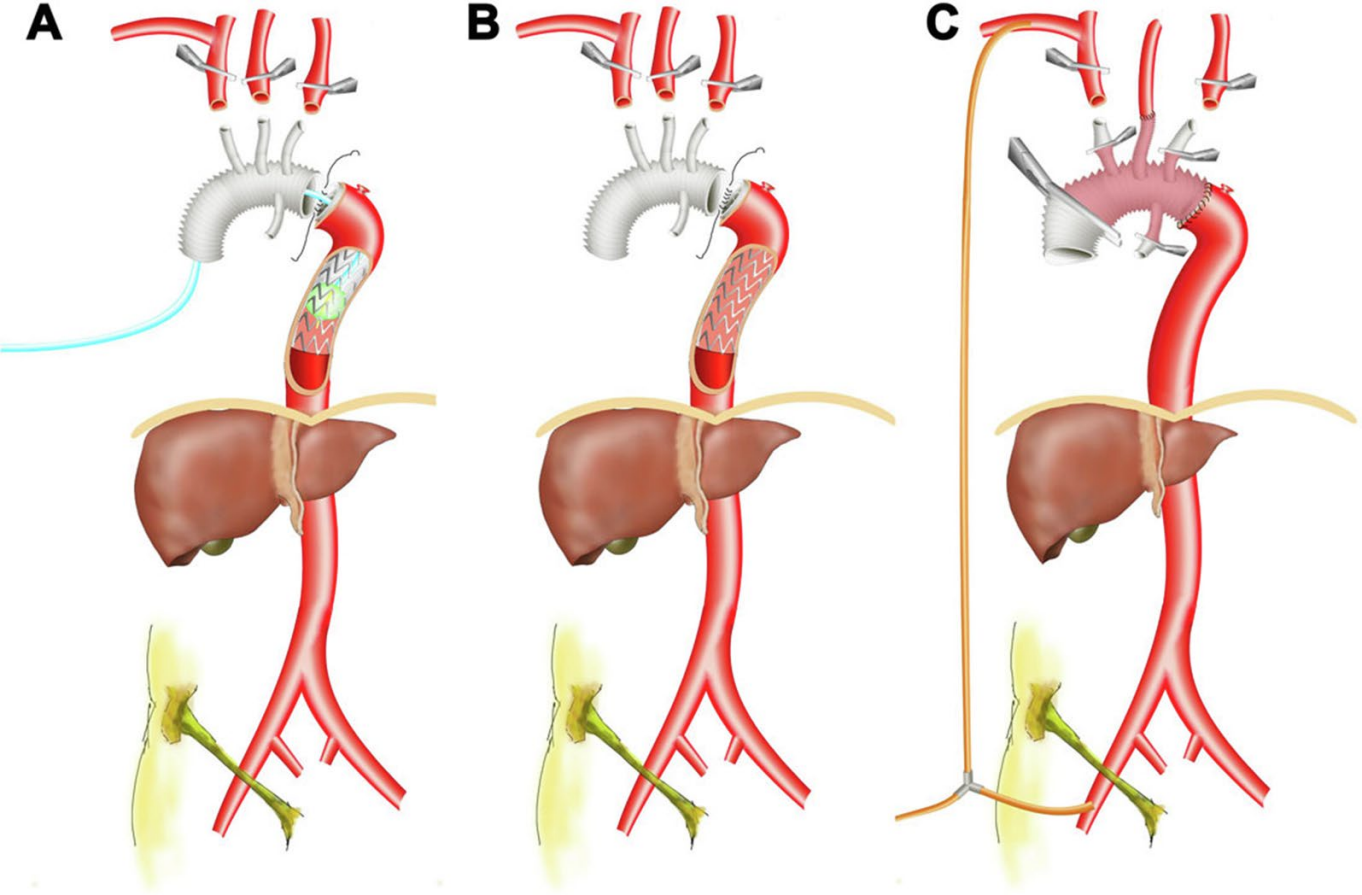
Illustration of surgical technique TAR with FET. **A** ABO. **B** Conventional TAR with FET technique. **C** Aortic reconstruction before rewarming. The standard cases were performed with right axillary cannulation for unilateral antegrade selective cerebral perfusion with femoral cannulation for retrograde systemic perfusion

In the surgical procedure, arterial cannulation was achieved using the right axillary and femoral arteries, delivered with a Y-connector from the single arterial line. After the nasopharyngeal temperature reached 28 °C, the femoral line was clamped and lower body CA was initiated, while unilateral antegrade selective cerebral perfusion was delivered via a right axillary line at the rate of 5–10 mL/kg per min with the proximal right innominate artery clamped. The descending aorta was implanted with a Cronus elephant trunk stent (diameter 26 or 28 mm, length 100 or 120 mm; Cronus, MicroPort Endovascular Shanghai Co., Ltd., Shanghai, China). The aortic balloon that fits the diameter of the elephant trunk stent graft, as well as the descending aorta that the graft sticks to (40 mL, Coda Balloon Catheter, Cook Incorporated, Bloomington, IN), was then deployed and inflated at the metal part of the stent graft. The aortic balloon also passed through a solid Gore sheath (16 or 18F W.L. Gore & Associates, Inc., Flagstaff, AZ) that was simultaneously held by hand and exerted sufficient force to counter the retrograde femoral perfusion when it started. The metal part of the stent elephant trunk could protect against injury of the aortic wall (because it is frail due to dissection or connective tissue disorders) as well as produce the static friction needed for the inflated aortic balloon. The lower body circulation resumed after this set up and end-to-end anastomosis of the aorta with a tetrafurcated graft (Terumo, Vascutek Limited, Renfrewshire, Scotland, UK) was performed under continuous perfusion. In the conventional method, the nasopharyngeal temperature must reach 26 °C or lower and anastomosis was performed during lower-body CA. Rewarming of the patient could be started when the common carotid artery was reconstructed and perfusion to both hemispheres was achieved [Fig. 1A–C].

The procedure is highly feasible and safe because the aortic balloon we used fits the diameter of the elephant trunk stent. When we inflate the balloon adequately in the descending aorta, it is simultaneously held in the solid Gore sheath to exert sufficient force to counter the femoral perfusion. The metal part of the elephant trunk stent could produce the static friction for the aortic balloon as well as prevent injury of the aortic wall. However, a larger amount of retrograde back flow from femoral perfusion may occur due to the large false lumen of the descending aorta. During our prior exploration, we learned first to use strong extra cardiac suction to vent the blood back to the cardiopulmonary bypass (CPB) circulation. If an even larger amount of back flow persisted, we correspondingly decreased the lower-body flow to create a clear surgical field, which is one of the reasons we set the target nasal temperature a relatively safe level (28 °C). In a few patients, we explored bilateral cerebral perfusion (both branched from the upper arterial cannula) to the right axillary artery and left common carotid artery, in which case, the target nasal temperature was set at 30 °C and CPB flow was set at 2/3 of total flow during the ABO so that both hemispheres had sufficient perfusion.

### Statistical analysis

The preoperative baseline characteristics and early outcomes were recorded according to the consensus statement from the International Aortic Arch Surgery Study Group [14]. Data from the blood examination were collected at the different time points to depict the trajectories of each examination as well as include them in the multivariate analysis.

The continuous variables are presented as median (lower quartile [Q1], upper quartile [Q3]), except for the quantity of blood transfusion, which is presented as mean ± standard deviation (SD). For univariate comparison, the normally distributed continuous variable (P > 0.05 in the Shapiro–Wilk test) was evaluated with the Student's t-test. The non-normally distributed continuous variable (P < 0.05 in the Shapiro–Wilk test) was evaluated with the Mann–Whitney U test, and categorical variables were compared using the χ2 test. Binary logistic regression was performed on clinical outcomes and the requirement of blood transfusion with the reasonable risk factors and checked for Hosmer–Lemeshow fitness before drawing conclusions. If the Hosmer–Lemeshow fitness failed, some risk factors were removed from consideration to make the fitness more than 0.05. Similar potentially overlapping factors could not be considered in the same comparison, so we picked only one in each model, such as being in the acute stage of aortic dissection (AD) (< 7 days of symptom onset) or being an emergency operation (surgery performed on admission day or next morning if admission at night), requiring longer postoperative conscious revival time or longer mechanical ventilation time, percentage of neutrophil and neutrophil count, and so on. We used total abdominal aorta involvement, which stands for AD reaching the distal end of the abdominal aorta with or without iliac involvement, to reflect the extension of AD on clinical results. As a result, the number of factors considered for each comparison did not exceed 32, which is reasonable because statistics suggests fewer than 1/10 of the cases (36 in our model). The statistical analysis was performed using SPSS (version 19.0, IBM-SPSS Inc, Armonk, NY).

## Results

### Patient baseline characteristics

Table 1 shows the preoperative and operative details. The median age of the ABO group and conventional operation group were 51 (42–58) and 47 (40–55) years and included 76.2% and 75.2% male patients, respectively. The patients had similar smoking history (56.9% vs. 56.5%), cardiovascular disease history (10.0% vs. 10.4%), and stroke history (8.5% vs. 10.0%). The most common indication for surgery was AD (94.6% vs. 96.1%) that occurred in the acute stage (76.2% vs. 78.7%). The aortic involvement was also similar, with the aortic arch (total or distal part only) at 100%, the aortic root at 43.1% and 42.1% proximally and either or both iliac arteries at 59.4% and 61.1% distally. Total arch replacement was performed on some patients with a thoracic endovascular aortic repair history due to proximal endoleak despite AD not involving a total aortic arch; the protocol was described in previous research [15]. With the ABO approach, the duration of lower-body CA was significantly shorter (5 [3–7] min vs. 17 [14–20] min, P < 0.001), with CA temperature rising from 25.4 (24.9–26.0) °C to 27.6 (27.1–28.0) °C accordingly (P < 0.001).

**Table 1:** Preoperative and operative details of consecutive patients who underwent TAR with FET technique

Variables	ABO (*n* = 130)	Conventional (*n* = 230)	Statistics	*P*
*Preoperative and operative details (contribution to EuroSCORE)*				
Age (years‡)	51 (42–58)	47 (40–55)	1.720*	0.087
Female (+1)	31 (23.9)	57 (24.8)	0.039*	0.843
COPD history (+1)	6 (4.6)	2 (0.9)	5.363*	0.021
Peripheral arterial disease history (+2)	11 (8.5)	13 (5.7)	1.054*	0.503
Stroke history (+2)	11 (8.5)	23 (10.0)	0.230*	0.632
Cardiac surgery history (+3)	2 (1.5)	15 (6.5)	4.584*	0.032
Preoperative Scr (μmol/L)	83.1 (71.7–105.8)	90.0 (71.7–114.3)	Non-normally distributed^†^	0.532
Scr > 200 μmol/L (+2)	1 (0.8)	6 (2.6)	1.474*	0.225
LVEF (%||)	51 (47–55.3)	50.5 (46–54)	Non-normally distributed^†^	0.187
Myocardial infarction in 90 days (+ 2)	9 (6.9)	11 (4.8)	0.725*	0.394
Pulmonary hypertension (+ 2)	1 (0.8)	3 (1.3)	0.216*	0.642
Emergency, operated on the day or next day upon admission (+ 2)	93 (71.5)	174 (75.7)	0.734*	0.392
Concomitant coronary artery bypass graft (+ 2)	18 (13.9)	30 (13.0)	0.046*	0.830
EuroSCORE	2 (3–4)	2 (3–4)	Non-normally distributed^†^	0.485
*Other preoperative details*				
Weight (kg)	75 (67.5–86)	75 (67.1–85)	Non-normally distributed^†^	0.839
Height (cm)	172 (169–176)	172 (167–176)	Non-normally distributed^†^	0.738
Heavy smoker	74 (56.9)	130 (56.5)	0.005*	0.941
CAD history	13 (10.0)	24 (10.4)	0.017*	0.896
*Aortic pathology*			0.424*	0.515
Aneurysm	7 (5.4)	9 (3.9)		
Aortic dissection	123 (94.6)	221 (96.1)		
Thoracic endovascular aortic repair history	10 (7.7)	13 (5.7)	0.578*	0.447
*Aortic dissection involvement*				
Root	53 (43.1)	93 (42.1)	0.033*	0.856
Ascending	105 (85.4)	208 (94.1)	7.381*	0.007
Arch	123 (100)	221 (100)	/	/
Total/ proximal	116 (94.3)	217 (98.2)	3.845*	0.050
Left subclavian artery or distal	7 (5.7)	4 (1.8)		
Thoracic descending	115 (93.5)	207 (93.7)	0.004*	0.951
Abdominal	99 (80.5)	196 (88.7)	4.350*	0.037
Iliac	73 (59.4)	135 (61.1)	0.100*	0.752
*Classification based on chronicity*				
Acute(< 7 d)	87 (66.9)	168 (73.0)	1.506*	0.220
Subacute(7–30 d)	23 (17.7)	20 (8.7)	6.392*	0.011
Chronic(> 30 d)	20 (15.4)	42 (18.3)	0.482*	0.488
*Operative details*				
Operation time (min)	379 (337–430)	395 (330–463)	Non-normally distributed^†^	0.445
CPB time (min)	175 (148–201)	162 (141–209)	Non-normally distributed^†^	0.152
Clamp (min)	114 (94–143)	109 (89–131)	Non-normally distributed^†^	0.034
CA time (min)	5 (3–7)	17 (14–20)	Non-normally distributed^†^	< 0.001
Require secondary CPB	5 (3.85)	29 (12.61)	7.457*	0.006
*Temperature change(°C)*				
Nasal temperature during CA	27.6 (27.1–28.0)	25.4 (24.9–26.0)	Non-normally distributed^†^	< 0.001
Rectal temperature during CA	28.7 (28–29.7)	28.1 (26.7–30.1)	Non-normally distributed^†^	0.015

Data are presented as median (Q1–Q3) or *n* (%)

*COPD* Chronic obstructive pulmonary disease, *EuroSCORE* European System for Cardiac Operative Risk Evaluation, *LVEF* left ventricular ejection fractions, *Scr* serum creatinine, *CA* circulatory arrest, *CPB* cardiopulmonary bypass

*t value;†: *χ*^2^ value; ‡EuroSCORE + 1 for 60 years and + 1 for every 5 years beyond 60; §EuroSCORE + 2 for preoperative serum creatinine more than 200 μmol/L; ||EuroSCORE + 1 for between 30 and 50% and + 3 for less than 30%

### In-hospital outcomes

Postoperative details and early clinical outcomes are listed in Table 2. Univariate comparison showed that 30-day mortality of the ABO group and conventional operation group were 4.6% and 7.8%, respectively (P = 0.241). The ABO group had fewer incidences of prolonged mechanical ventilation (> 72 h), severe lung infection, hepatic injury, and acute kidney injury (AKI). Length of intensive care unit (ICU) stay and postoperative in-hospital stay was similar as well. Despite that the European System for Cardiac Operative Risk Evaluation (EuroSCORE) for both groups was 3 (2 to 4) (P = 0.485), several differences exist. The ABO group was older, with much less secondary cardiac surgery history, difference in AD involvement, and unevenly distributed CPB time. The aorta cross-clamp periods of ABO were longer (114 [94–143] min vs. 109 [89–131] min, P = 0.034) but less secondary CPB was performed (3.9% vs. 12.6%, P = 0.006). Multivariate analysis was performed to find detailed risk factors for each clinical outcome in all consecutive cases. Risk factors for 30-day mortality were heavy smoker (odds ratio, OR, 10.898), chronic obstructive pulmonary disease (COPD) history (OR 10.198), emergency operation (OR 63.887), preoperative percentage of neutrophils (OR 1.123 per %), and CPB time (OR 1.010 per min) [Table 3].

**Table 2:** Postoperative details and early clinical outcomes of consecutive patients who underwent TAR with FET technique

Variables	ABO (n = 130)	Conventional (n = 230)	Statistics†	P
30-day mortality	6 (4.6)	18 (7.8)	1.376	0.241
Conscious revival (h)	8.1 (4.9–13.8)	11.8 (7.8–19.0)	non-normally distributed	< 0.001
Delayed conscious revival (> 12 h)	38 (29.2)	103 (44.8)	8.431	0.004
Mechanical ventilation (h)	18.7 (13.3–36.9)	21.5 (13.5–47.0)	non-normally distributed	0.217
Prolonged mechanical ventilation (> 72 h)	11 (8.5)	42 (18.3)	6.535	0.012
ICU stay (h)	90.5 (59.3–132.8)	86.4 (58.7–134.2)	non-normally distributed	0.380
Postoperative in-hospital stay (d)	11 (8–14)	11 (9–14)	non-normally distributed	0.321
Postoperative serum creatinine (day 1)	107.4 (85.8–137.8)	168.2 (69.7–202.8)	non-normally distributed	0.002
AKI (serum creatinine > 200 μmol/L)	30 (23.1)	82 (35.7)	6.128	0.013
Oliguria or anuria	10 (7.7)	22 (9.1)	0.360	0.549
Continuous renal replacement therapy	10 (7.7)	23 (10.0)	0.531	0.466
Stroke	4 (3.1)	9 (3.9)	0.167	0.683
Temporal paraplegia	3 (2.3)	10 (4.4)	0.993	0.319
Delirium	4 (3.1)	15 (6.3)	1.972	0.160
Acute hepatic injury	16 (12.3)	64 (27.8)	11.572	0.001
Sub-acute hepatic injury	55 (42.3)	85 (37.0)	1.001	0.317
Platelet rebound‡	90 (69.3)	190 (82.6)	8.600	0.003
Severe lung infection	3 (2.3)	18 (7.8)	4.604	0.032
Total chest tube drainage of 0–3 postoperative day (mL)§	1040 (720–1260)	1140 (860–1460)	non-normally distributed	0.002
Percentage of > 1500 mL	18 (13.9)	53 (23.0)	4.438	0.035
Operation day	380 (280–480)	460 (360–685)	non-normally distributed	0.760
Postoperative day 1	250 (200–330)	330 (240–450)	non-normally distributed	0.001
Postoperative day 2	170 (110–240)	225 (160–310)	non-normally distributed	< 0.001
Postoperative day 3	160 (80–230)	190 (135–270)	non-normally distributed	< 0.001
Cost of medical bill (USD)	25,463 (21,889–29,627)	24,914 (22,077–29,378)	non-normally distributed	0.298
Less than 20,000	10 (7.7)	24 (10.4)	0.903	0.637
20,000 to 30,000	89 (68.5)	157 (68.3)		
More than 30,000	31 (23.9)	49 (21.3)		
**Blood product transfusion (expressed as percentage of usage and average quantity among used)**				
Red blood cell transfusion (U)	7.8 ± 6.7 83 (63.9)	8.5 ± 8.7 174 (75.7)	non-normally distributed (5.668)	0.581 (0.017)
Intraoperative	4.8 ± 3.1 55 (42.3)	5.9 ± 4.9 117 (50.9)	non-normally distributed (2.440)	0.259 (0.118)
During CPB	4.4 ± 1.9 43 (33.1)	4.8 ± 2.6 78 (33.9)	non-normally distributed (0.026)	0.612 (0.872)
After CPB	3.6 ± 3.2 21 (16.2)	4.9 ± 4.3 65 (28.3)	non-normally distributed (6.696)	0.056 (0.010)
Postoperative	5.9 ± 4.8 65 (50.0)	6.0 ± 7.1 132 (57.4)	non-normally distributed (1.831)	0.741 (0.176)
Plasma transfusion (ml)	770.7 ± 549.0 82 (63.1)	997.2 ± 856.6 141 (61.3)	non-normally distributed (0.111)	0.011 (0.739)
Intraoperative	550.7 ± 392.0 71 (54.6)	675.8 ± 369.2 124 (53.9)	non-normally distributed (0.017)	0.001 (0.898)
Postoperative	573.8 ± 406.7 42 (32.3)	778.0 ± 798.3 73 (31.7)	non-normally distributed (0.012)	0.153 (0.912)
Platelets transfusion (U)	1.5 ± 0.9 102 (78.6)	1.7 ± 1.5 189 (82.2)	non-normally distributed (0.739)	0.338 (0.390)
Intraoperative	1.2 ± 0.5 93 (71.5)	1.2 ± 0.9 172 (74.8)	non-normally distributed (0.450)	0.734 (0.502)
Postoperative	2.2 ± 1.2 18 (13.9)	2.2 ± 2.0 51 (22.2)	non-normally distributed (3.718)	0.304 (0.054)

*ABO *Aortic balloon occlusion, *TAR* total aortic arch replacement, *FET* frozen elephant trunk, *CPB *cardiopulmonary bypass, *USD* United States dollar, *AKI* acute kidney injury

*Data are expressed as median (Q1–Q3) or *n*(%), except for the quantity of blood transfusion, which is presented as mean ± standard deviation

^†^Statistical values for normally distributed continuous variable is t value, for categorical variables is χ2 value, or is noted as non-normally distributed continuous variable

^‡^Platelet rebound stands for the last re-examination of platelet count during in hospital stay was higher than the preoperative level

^§^Total chest tube drainage of 0–3 postoperative day (per 100 mL) was considered in the multivariable analysis for postoperative bleeding as it is a direct factor for blood loss

**Table 3:** Multivariable analysis of risk factors for clinical outcomes

Risk factors (Hosmer–Lemeshow fitness*)	Odds ratio	95% confidence interval	*P*
*30-day mortality (0.189)*			
Heavy smoker	10.898	1.974–60.151	0.006
COPD history	10.198	1.096–94.915	0.041
Emergency operation	63.887	4.828–845.303	0.002
Preoperative leukocyte count (10^9^/L)	(1.109)	(0.942–1.305)	(0.213)
Preoperative percentage of neutrophils (%)	1.123	1.020–1.237	0.018
CPB time (min)	1.010	1.005–1.015	< 0.001
*Continuous renal replacement therapy (0.401)*			
COPD history	7.965	1.079–58.783	0.042
Emergency operation	7.972	1.003–58.709	0.049
Preoperative Scr (μmol/L)	1.011	1.001–1.021	0.028
Preoperative leukocyte count (10^9^/L)	1.191	1.015–1.399	0.032
Preoperative percentage of neutrophils (%)	(0.983)	(0.920–1.050)	(0.614)
Preoperative D-dimer (μg/ml)	1.088	1.007–1.017	0.032
CPB time (min)	1.013	1.008–1.018	< 0.001
*Acute kidney injury (0.709)*			
Weight (kg)	1.046	1.006–1.087	0.023
Height (cm)	0.954	0.916–0.993	0.022
Conventional group	2.493	1.025–6.061	0.044
Concomitant CABG	3.419	1.142–10.233	0.028
Preoperative Scr (μmol/L)	1.049	1.033–1.065	< 0.001
Preoperative hemoglobin (g/L)	1.044	1.010–1.078	0.010
Preoperative platelet count (10^9^/L)	0.922	0.985–0.999	0.032
Postoperative neutrophil count 0† (10^9^/L)	1.200	1.081–1.332	0.001
Postoperative platelet count 0 (10^9^/L)	0.989	0.978–1.000	0.044
*Acute hepatic injury (0.953)*			
Conventional group	2.326	1.230–4.399	0.009
Preoperative AST (U/L)	1.031	1.005–1.057	0.021
CPB time (min)	1.008	1.003–1.014	0.002
Postoperative platelet count 0 (10^9^/L)	0.989	0.983–0.996	0.002
Postoperative leukocyte count 0 (10^9^/L)	1.094	1.019–1.176	0.014
Postoperative percentage of neutrophils 0 (%)	0.886	0.829–0.946	< 0.001
Postoperative Scr (μmol/L)	1.010	1.004–1.015	< 0.001
*Temporal paraplegia (0.328)*			
Total abdominal aorta involvement	10.147	0.755–136.293	0.080
Preoperative Scr (μmol/L)	1.023	0.996–1.052	0.099
*Delirium (1.000)*			
Postoperative mechanical ventilation (h)	1.033	1.010–1.057	0.005
Postoperative FDP (μg/mL)	1.067	1.001–1.136	0.045
*Delayed conscious revival (> 12 h) (0.526)*			
Age (yrs)	1.048	1.019–1.078	0.001
Weight (kg)	1.027	1.005–1.050	0.015
Conventional group	2.521	1.475–4.309	0.001
Female	2.512	1.108–5.695	0.027
Preoperative ALT (U/L)	1.011	1.001–1.022	0.031
Postoperative Scr (μmol/L)	1.007	1.000–1.014	0.043
*Prolonged mechanical ventilation (> 72 h) (0.342)*			
CPB time (min)	1.011	1.006–1.016	0.001
*Severe lung infection (0.714)*			
Heavy smoker	80.126	2.161–2971.041	0.017
COPD history	48.864	1.337–1786.397	0.034
CPB time (min)	1.014	1.005–1.024	0.002
Postoperative Scr (μmol/L)	1.011	1.000–1.023	0.049
*Platelet rebound‡ (0.106)*			
Conventional group	2.046	1.130–3.704	0.018
Postoperative platelet count 0 (10^9^/L)	0.986	0.979–0.992	< 0.001
Postoperative leukocyte count 0 (10^9^/L)	1.121	1.034–1.215	0.006
Postoperative percentage of neutrophils 0 (%)	(0.992)	(0.928–1.060)	(0.809)
RBC transfusion total (U)	0.907	0.836–0.984	0.019
Plasma transfusion total (ml)	0.999	0.999–1.000	0.038
Final re-exam of platelet count time (d)	1.163	1.086–1.244	< 0.001
*Sub-acute hepatic injury (0.826)*			
Female	0.317	0.107 to 0.937	0.038
Postoperative ALT1§ (U/L)	1.008	1.001 to 1.015	0.032
Postoperative AST1 (U/L)	0.996	0.992 to 1.000	0.038
*Cost less (< 20,000 USD) (0.804)*			
Total abdominal aorta involvement	0.250	0.109–0.575	0.001
CPB time (min)	0.978	0.965–0.990	0.001
*Cost more (> 30,000 USD) (0.699)*			
Age (yrs)	1.034	1.001–1.069	0.046
Weight (kg)	1.033	1.006–1.061	0.017
Cardiac surgery history	6.211	1.828–21.103	0.003
Preoperative Scr (μmol/L)	1.011	1.004–1.019	0.004
Preoperative percentage of neutrophils (%)	0.956	0.917–0.996	0.031
Preoperative D-dimer (μg/mL)	1.062	1.004–1.124	0.036
CPB time (min)	1.007	1.003–1.011	< 0.001

*ALT* Alanine transaminase, *AST* aspartate transaminase, *COPD* chronic obstructive pulmonary disease, *CPB* cardiopulmonary bypass, *FDP* fibrinogen degradation product, *RBC* red blood cell count, *Scr*serum creatinine, *USD* United States dollar, *CAD* coronary artery disease

^*^Each multivariable analysis of risk factors can be accepted if the *P* value of Hosmer–Lemeshow fitness was more than 0.05

^†^All the postoperative blood test 0 stands for the immediate reexamination time point when the operation ended and transferred to ICU

^‡^Platelet rebound stands for the last re-examination of platelet count during in hospital stay was higher than the preoperative level

^§^All the postoperative blood test 1 stands for the reexamination time point of postoperative day 1

### Renal system risk factors

Postoperative AKI was defined as serum creatinine (Scr) increased by more than 1.5 times baseline values, which was Scr > 200 μmol/L. Being in the conventional group was a risk factor for AKI (OR 2.493), which reflected that longer lower body CA, increased AKI. Higher preoperative hemoglobin was also a risk factor for AKI (OR 1.044 per g/L), which suggested that the relatively lowered operative hemoglobin content during CPB could not be well tolerated by the kidneys. Both factors reflected the perfusion operative status of the kidney, which suggested that Scr was a very sensitive indicator of the operative renal perfusion. The magnitude of the operative platelet-counts' decrease was reported to correlate with the severity of AKI in previous research [16, 17]. In our analysis, higher platelet counts preoperatively (OR 0.922 per 109/L) and postoperatively (OR 0.989 per 109/L) were shown to decrease the incidence of AKI. Creatinine generation is proportional to the body muscle weight, thus higher body weight may increase Scr and the incidence of AKI (OR 1.046 per kg). Continuous renal replacement therapy (CRRT) is the treatment for deteriorating renal injury and patients manifesting oliguria were given CRRT (Table 2). In addition to higher preoperative Scr (OR 1.011 per μmol/L), the risk of requiring CRRT is much higher from longer CPB time (OR 1.013 per min). If CPB time becomes excessively long, the kidney will be continuously exposed to a relatively ischemic state, which is bound to increase the need for CRRT. The protective effect of ABO was limited, which failed to deliver statistical significance in reducing the need for CRRT.

### Hepatic system risk factors

**Fig. 2:**
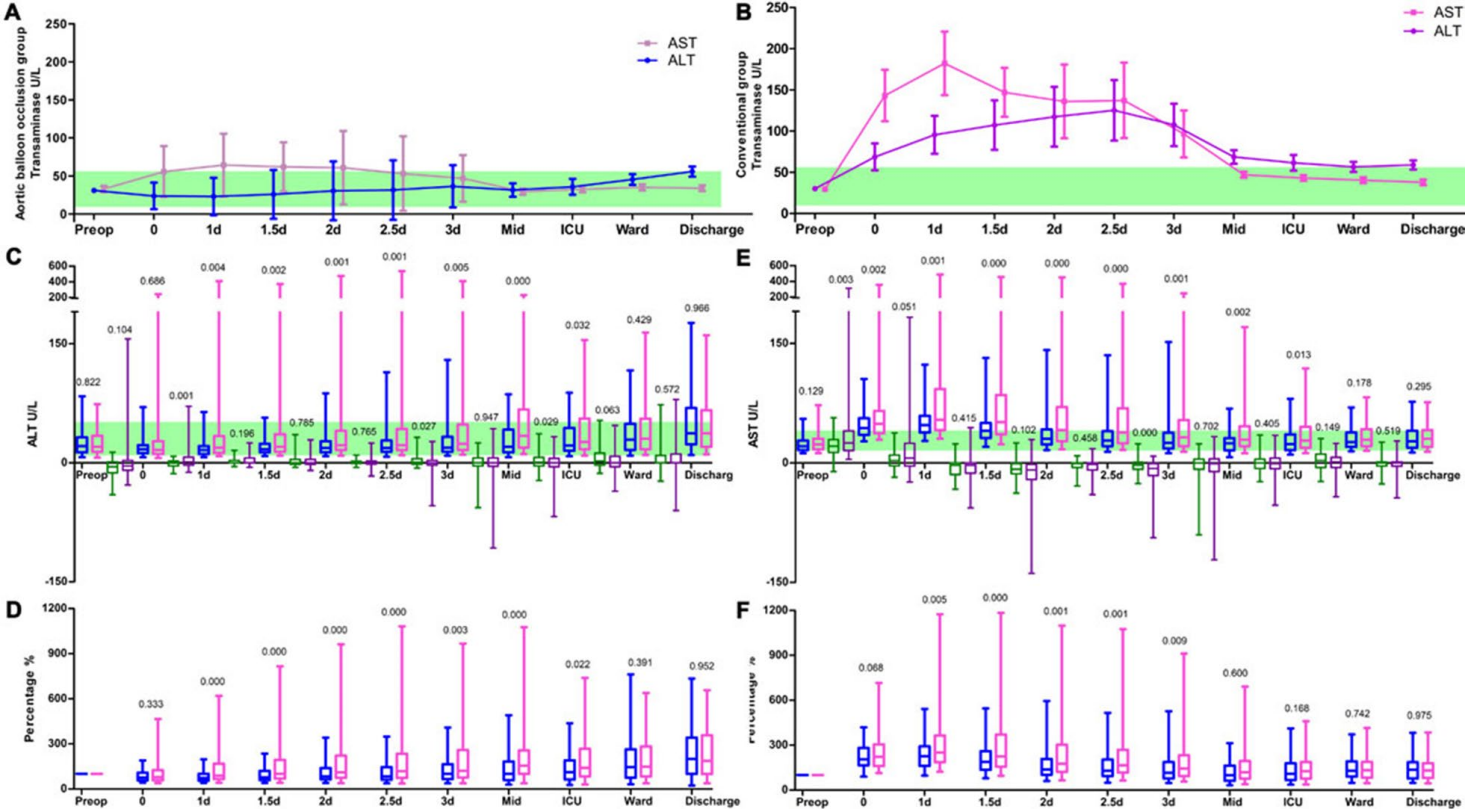
Trajectory of transaminase in two group of patients with TAR with FET. **A** Trajectory of alanine transaminase (ALT) and aspartate transaminase (AST) in the ABO group (*n* = 130). **B** Trajectory of ALT and AST in the conventional TAR with FET group (*n* = 230). **C** Box plot of ALT. **D** ALT ratio. **E** Box plot of AST. **F** AST ratio. Data points of A and B are represented by mean with standard error of mean bars. There are 4 types of box plots in C and E in each time points, which are the examination levels of the ABO group (blue), the conventional group (pink), difference of levels between the points in the ABO group (green), and in the conventional group (purple), respectively. The green band represents the referred normal range of the examination. D and F are the examination levels compared to preoperative values. *P* value is represented above the standard error of mean bars or the boxes. Time points were defined as before the operation (Preop), the time when the operation ended and transferred to ICU (0), twice on postoperative days (POD) 1 to 3 (1 d: POD1 morning; 1.5: POD1 afternoon; 2 d: POD2 morning; 2.5: POD2 afternoon; 3: POD3 morning), additional point of ICU stay if the patient stayed in there more than 4 d (Mid), the last examination before transfer from the ICU (ICU), the first examination after transfer from the ICU to the ward (Ward), and the last examination before discharge (Discharge). The rationale for the choice of these time points is to include both time-matched (postoperative days 1 to 3) and event-matched (transfer out of ICU, discharge) comparison

Hepatic injury is defined as hepatic transaminase increased by > 1.5 times baseline values for 48 h. We recorded the trajectories of alanine transaminase (ALT) and aspartate aminotransferase (AST) in Fig. 2. AST was considerably higher in the first 3 postoperative days and fell to normal range before discharge. ALT rose steadily rise in the ABO group. ALT and AST were very sensitive in reflecting lower body perfusion. The levels of ALT and AST were much higher in the conventional group, making presence in that group an independent risk factor for hepatic injury (OR 2.326). Our results showed that AST was much more sensitive than ALT, similar to previous findings [18–20]. Preoperative AST, but not ALT, is an independent risk factor for hepatic injury (OR 1.031). Acute hepatic injury was also risked from longer CPB time (OR 1.008 per min) and higher postoperative Scr (OR 1.010 per μmol/L). The partial overlap of acute hepatic injury and AKI suggested that they both posed risks from insufficient lower-body perfusion but manifested in different subpopulations due to differences in other innate risk factors. The liver is the source of blood coagulation factors, which would explain why a higher AST level was related to an increase in red blood cell (RBC) and plasma transfusion requirements [Additional file 1: Table 1].

The AST level must have recovered to a normal range before discharge. Unlike AST, the ALT level tended to grow progressively, although some patients experienced fluctuating levels in the first 3 postoperative days. The ALT level became higher than the preoperative value before discharge. This slow and steady elevation of ALT suggested that it reflected the mid-term sub-acute hepatic injury. Sub-acute hepatic injury is less likely to appear in female patients (OR 0.317). A higher AST level on postoperative day 1, which is the indicator for acute hepatic injury, is negatively related to sub-acute hepatic injury (OR 0.996 per U/L).

### Nervous system risk factors

The risk factors for most clinical outcomes could be established, except for postoperative stroke and temporary paraplegia, which were relatively lower than other clinical adverse outcomes and could only be compared with a larger number of patients. If we loosened the P value from < 0.05 to < 0.1, temporary paraplegia was a positive risk from total abdominal aorta involvement and preoperative Scr, both of which suggested that a longer extension of AD and possibly more segmental branches of the aorta supplying the spinal cord were involved. The postoperative conscious revival time from anesthesia was significantly shortened by application of ABO. Even though both groups received continuous cerebral perfusion, the nadir nasal temperature was lower with significantly longer cooling and rewarming time in the conventional group, which made being in that group a risk factor for prolonged conscious revival time (> 12 h) (OR 2.521). Older age (OR 1.048 per year) and female gender (OR 2.512) also tended to delay conscious revival. Most anesthetics metabolized through the liver and some through the kidney, which partially explained why the factors of preoperative ALT (OR 1.011 per U/L), postoperative Scr (OR 1.007 per μmol/L), and greater weight (because more anesthetics were used, OR 1.027 per kg) increased the risk of delayed conscious revival. The patient had to revive from anesthesia before withdrawal of mechanical ventilation. Even if the patient revived earlier, longer mechanical ventilation time could increase the risk of delirium (OR 1.033), a status of incomplete of consciousness that would require antipsychotics. It could be partially explained by cerebral hypoxia observed in previous research [21]. Higher postoperative fibrinogen degradation product (FDP) was also a risk for delirium (OR 1.067).

### Respiratory system risk factors

Prolonged mechanical ventilation was solely risked from longer CPB time (OR 1.011 per min). Longer CPB meant more operative damage and more compromised immune system which would be more susceptible to severe inflammation. Severe lung infection meant prolonging mechanical ventilation despite prolonged strong antibiotics usage, in which situation surgical or endoscopic intervention was required. Its risk factor included patients' basic lung function (heavy smoker, OR 80.126; and COPD history, OR 48.864), as well as longer CPB time (OR 1.014 per min). Higher postoperative Scr (OR 1.011 per μmol/L) posed a risk for severe lung infection, although ABO did not directly relate to severe lung infection.

### Blood routine tests and transfusion risk factors

**Fig. 3:**
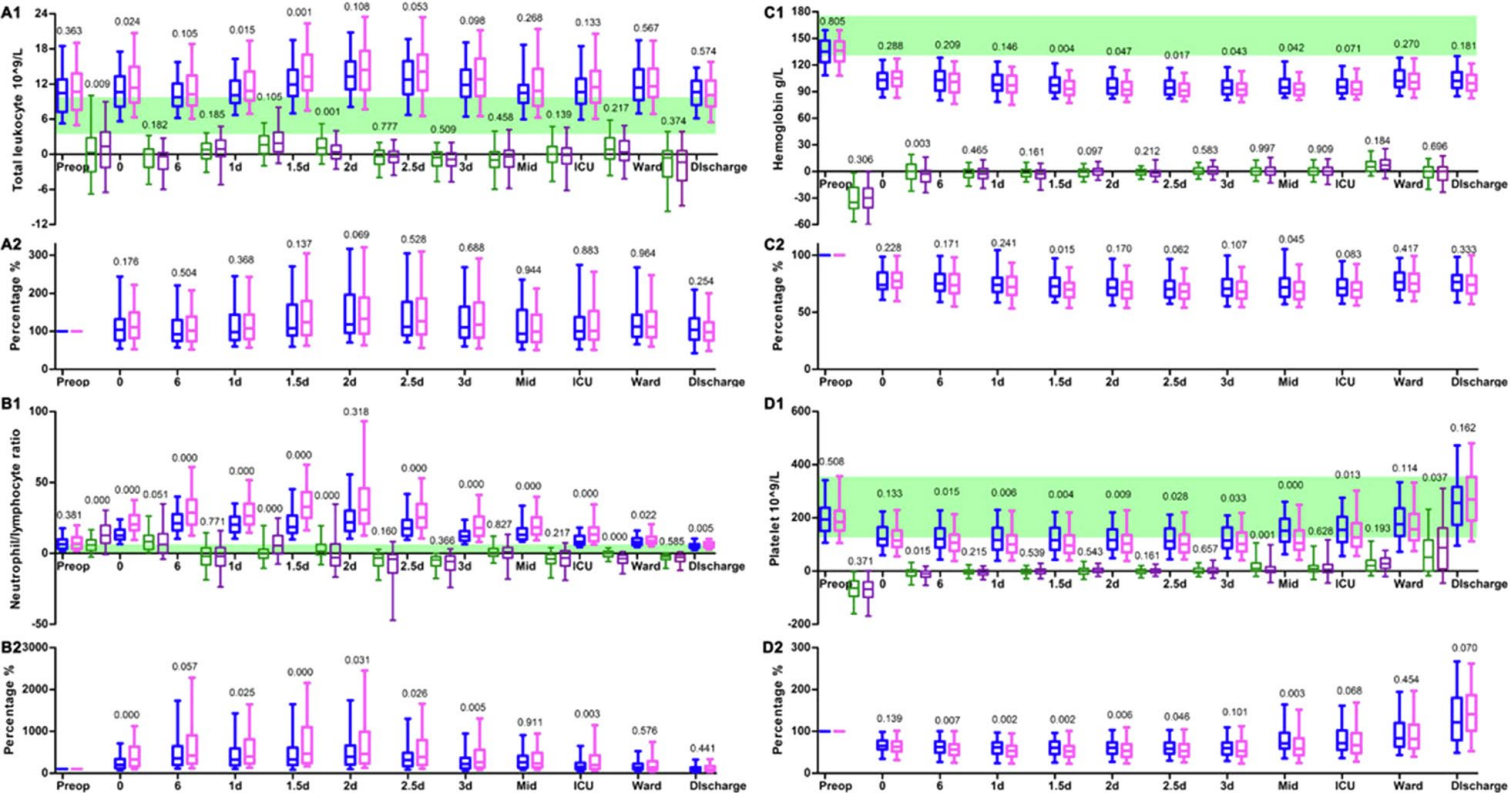
Blood routine tests of the ABO group (*n* = 130) and the conventional TAR with FET group (*n* = 230). **A** Leukocyte count: A1, value; A2 ratio. **B** Neutrophil/lymphocyte ratio: B1, value; B2 ratio. **C** Hemoglobin: C1, value; C2 ratio. **D** Platelet count: D1, value; D2 ratio. There are 4 types of box plots in A1, B1, C1, and D1 in each time points. A2, B2, C2, and D2 are the examination levels compared to preoperative values. *P* value is represented above the boxes

Total leukocyte count and neutrophil/lymphocyte ratio (NLR), which are inflammatory indicators, are represented in Fig. 3A, B. Multivariate analysis showed a higher preoperative total leukocyte or neutrophil count was a risk factor for AKI, CRRT, hepatic injury, and 30-day mortality.

Blood hemoglobin was at similar levels in both groups preoperatively. During CPB, both groups received similar quantities of RBC transfusion, suggesting that blood loss due to the continuation of CPB was similar. After CPB during the operation, the ABO group required much less RBC transfusion. As a result, ABO reduced total RBC usage [Fig. 3C and Table 2].

Univariate comparisons were not accurate because many other factors affected transfusion. Multivariate analysis was performed on events if transfusion of RBC, plasma, or platelet was required (> 0 package) and if the requirement for transfusion was large (reached or was more than 3 packages, 6 U of RBC, 600 mL of plasma, or 3 U of platelet). The result showed that cardiac surgery history was a risk factor for operative RBC transfusion (OR 7.703) and large operative RBC transfusion (OR 8.760). Female gender, older age, emergency operation, higher preoperative leukocyte or neutrophil count, postoperative mechanical ventilation time, and longer CPB time were risk factors that increased RBC transfusion. Being in the conventional group was a risk factor for large operative RBC transfusion after CPB (OR 25.520) but not during CPB, large total operative RBC transfusion (OR 3.554), and large total RBC transfusion during the in-hospital stay (OR 2.134). Large postoperative transfusion was also risked from postoperative AST (OR 1.004 per U/L) and postoperative Scr (OR 1.011 per μmol/L), suggesting that large transfusion may be the cause or caused from postoperative hepatic injury or AKI. ABO did not reduce the plasma and platelet transfusion requirements, according to multivariate analysis. Longer CPB time was the most important factor affecting nearly all aspects of blood product transfusion. Longer post-operative mechanical ventilation, cardiac surgery history, female gender, and aortic pathology (total abdominal aorta involvement) were strong factors positively related to blood product transfusion [Additional file 1: Table 1].

### Coagulation system risk factors

The coagulation factors were contained in the plasma. The ABO group required less plasma transfusion during the operation. Platelet transfusion was similar between the groups [Table 2]. The trajectory of platelet count is shown in Fig. 3D and its pattern is similar to previous researches [16, 22, 23]. During CPB, the platelets were severely diluted and damaged and about 80% of patients required platelet transfusion to control bleeding from the suturing properly. Even with the plasma and platelet transfusion, the bleeding from the chest tube was still relatively large in the earlier postoperative days [Table 2 and Additional file 1: Fig. 1]. Unlike RBC content, which stayed at a low level after the operation and most patients were still in an anemic state before discharge, the platelet count showed evident rising tendency at some point of the recovery process, which mostly appeared around postoperative day 3. Previous research also observed this phenomenon and argued that this process is indicative of a persistent platelet activation and aggregation in the first three postoperative days [16, 22, 23]. In the present study, the platelet count delayed to show the rising tendency in the conventional group because those patients experienced a larger quantity of blood loss from the chest tube in the first 3 preoperative days. There were 69.2% of patients in the ABO group and 82.6% in the conventional group (P = 0.003) whose last re-examination of platelet count during in hospital stay was higher than the preoperative level (platelet rebound). Multivariate analysis showed that a higher postoperative platelet count on operation day (0) would reduce the incidence of platelet rebound (OR 0.986 per 109/L) probably due to negative feedback. The later the re-examination of the platelet count is, the higher platelet count would rise (OR 1.163 per day), suggesting its tedency to rise continuously during our observation window. A higher platelet count was also an indication of inflammation; thus, being in the conventional group (OR 2.046) and having a higher leukocyte count (OR 1.121 per 109/L) were also risk factors for persistent platelet activation and aggregation. The final platelet count before discharge was apparently negatively related to the quantity of RBC (OR 0.907 per U) and plasma (OR 0.999 per mL) transfusion because they diluted the platelet concentration.

Pre- and postoperative D-dimer and FDP are shown in Additional file 1: Fig. 2. Higher preoperative FDP is a risk factor for RBC transfusion during CPB. Higher postoperative FDP was a risk factor for large platelet (≥ 3U) transfusion [Additional file 1: Table 1].

### Risk factors tended to increase cost

The overall medical bill for the ABO group and conventional group was 25.5 (21.9–29.6) vs. 24.9 (22.1–29.4) kUSD, respectively (P = 0.298). Old age (OR 1.034 per year), greater body weight (OR 1.033 per kg), cardiac surgery history (OR 6.211), higher preoperative Scr (OR 1.011 per μmol/L), higher preoperative D-dimer (OR 1.062 per μg/mL) and longer CPB time (OR 1.007 per min) were risk factors for increased cost (> 30 kUSD).

## Discussion

The ABO technique is a perfusion strategy of the TAR and FET technique that featured consistently shortened lower-body CA that subsequently raised the target nadir temperature setting point during CA, which provided end-organ protection during the operation. In multivariate analysis, the ABO technique was shown to prevent postoperative AKI, hepatic injury, delayed conscious revival, and large quantity of RBC transfusion. Even with the ABO technique as a protective factor, the TAR with FET still entailed by different risks for serious complication during the routine postoperative course. Although freeing the patient from major complications was the major goal, we usually felt that most postoperative complications occurred randomly and the risk factors behind them were overlooked. Thus we need clear understanding of how the commonly recognized risk factors matched up with different clinical outcomes. Before the operation, we always evaluated whether the patient would benefit more from the operation than suffer from the susceptible risks. The study of the mechanisms of serious complication in the new setting of ABO would be helpful in the treatment and prevention of complications from aortic operations.

Only a trend showing that ABO reduced 30-day mortality could not reach statistical significance. We found several risk factors for in-hospital mortality, but the only adjustable factor for the surgeon was longer CPB time and the other innate factors contributed to the incidence of mortality, which the surgeon should be aware of prior to the operation. This result suggests that CPB time is the most important factor related to an increase in 30-day mortality because it increases the risk by the minute, which outweighs the other factors if the procedure lasts longer. ABO, however, was not significant in terms of reducing mortality. The most important risk factor causing postoperative complications was longer CPB time, affecting nearly all aspects, particularly 30-day mortality and CRRT. Application of ABO decreased the incidence of AKI and hepatic injury and shortened postoperative conscious revival time in the overall population of our cohort, but its effect was limited and might not translate into less 30-day mortality or CRRT. The preoperative Scr level largely reflected the profound development of AD that might have caused renal malperfusion. A higher preoperative Scr level was one of the risk factors for AKI, need for CRRT, delayed conscious revival, severe lung infection, and increased medical bills. Postoperative Scr level indicated operative damage. Higher postoperative Scr level was one of the risk factors for increased RBC and platelet transfusion. Higher postoperative platelet count, which was an indication of less operative damage, was an independent predictor of less AKI and hepatic injury. Although the mutual indication in AKI and platelet count was described in a previous research [16, 17], the injury to kidney, liver, and blood cells in our patients were ultimately caused from the operation. During surgery, ABO could provide more reliable end-organ perfusion and higher temperature which prevented prolonged systemic cooling and rewarming. Such improvements might be crucial in preventing postoperative complications for patients who already carry great risks and are on the margin of developing complications.

The NLR is recognized as a marker of inflammation associated with poor outcomes after cardiac surgery [24] and aortic surgery [25–27]. In terms of postoperative care, neutrophil count would naturally elevate because of the unwanted inflammation resulting from the stimulation of the cardiac surgery and rise even higher if bacterial or fungal infection occurred, but the lymphocyte count, which is the indicator of compromised immune system, would naturally decrease. The lymphocyte count is much lower in frail patients resulting from both their preoperative conditions and the operative damage. The rise of the neutrophil count and fall of lymphocyte count make NLR the much more sensitive indicator of the overall condition of patients in the ICU than any leukocyte count alone does. As a result, the total leukocyte count did not show statistical difference, whereas the NLR rose much higher in the conventional group. Their alteration pattern of total leukocyte count and NLR suggested that their inflammatory status was much more severe in the earlier postoperative days in the ICU because of the acute operative stimulus, but evidently recovered to normal values before discharge. Considering that the preoperative total leukocyte counts were at high levels in both groups, we believe this high leukocyte count was caused by the acute onset of AD; even if the leukocyte count was high, the patients still had to be operated on in this emergency situation.

When CPB started, the bypass prime solution was mixed with the blood of the patient's own circulation, which created the risk of excessive hemodilution. CPB would then damage RBC and trigger transfusion if the blood hemoglobin fell below 70 g/L. After CPB, the RBC transfusion threshold was 80 g/L. We followed the RBC transfusion guideline of 80 g/L according to previous research, which suggested that such a lower level could achieve acceptable early outcomes [28]. As a result, the quantity of transfusion is quite large, similar to a previous report [29] and successfully maintained the postoperative blood hemoglobin level. Compared to the relatively slow recovery of hemoglobin, the platelet counts showed a rapid rebound during the ICU stay. In addition to this commonly recognized factor, we found several hemodynamic factors in the multivariate analysis of transfusion. RBC and platelet transfusion was negatively related to the preoperative level of hemoglobin and platelet count, as well as to body mass. Patients with higher weight and more blood cell concentration had more blood cell reserve, and if the operation exerted similar damage to the blood cells, they were less likely to require transfusion. On the contrary, the plasma transfusion requirement was positively related to body mass, because the CPB could quickly eliminate nearly all blood coagulation factors and the patients who weighted higher required more plasma to replenish coagulation factors. Another hemodynamic factor, total chest tube drainage in postoperative day 1–3 was strongly related to postoperative transfusion blood directly lost from the chest tube.

The most obvious limitation of this study is the inaccurate univariate comparisons for the continuous variables; thus, we also presented the blood examination trajectories with additional figures, in which they were normalized to the preoperative baseline. We only presented the data within only a short time span (since the invention of the technique in Aug 2017). Even with the same standard of postoperative care, differences of preoperative characteristics between the groups weakened the univariate comparisons. The preoperative characteristics also differed from other centers in several aspects; in particular our patients were much younger and had no abdominal malperfusion or preoperative shock, making our results difficult for direct comparison to other centers [30]. In addition, the shorter circulatory arrest (CA) time in the control group may have contributed to the lack of significant differences in some aspects between them and the patients in the ABO group. Based on the fact that Fuwai Hospital is one of the world's largest centers for cardiac surgery, and its total number of procedures and operating techniques for aortic dissection are at an international advanced level, this is consistent with the 17 min CA time of patients in the non-ABO group in this paper, however, the CA time in general hospitals is much longer than 17 min, so this paper would cause selection bias and weaken the differences between the ABO group and the non-ABO group in various aspects. In the future, it may be possible to observe whether patients who received the ABO technique have a better prognosis by comparing the differences between the two groups in other hospitals or by performing long-term follow-up of the two groups in our center.

## Conclusions

Aortic surgery with CA carries greater risks for hepatic, renal, and hematological injury than other routine cardiac operations. The ABO technique could serve as an important operative protective factor that improves the biochemical tests and the recovery process during in-hospital stay, although it did not translate into less CRRT or mortality. However, each complication was specifically affected by different preoperative and operative factors. Thus, the ABO technique cannot be regarded as the ultimate tool to prevent complications. We still believe that the continuous perfusion that ABO provided was crucial in diminishing the operative injury for frail or risky patients who might not survive the operation. Identification of the risk factors for adverse clinical outcomes is crucial for improving protocols to optimize perioperative care.

## Supplementary Information

**Additional file 1**. **Supplementary Figure 1**: Postoperative chest tube output of the ABO group (left column of each point, n = 130) and the conventional TAR with FET group (right column of each point, n = 230). Each bar represents the percentage of chest tube remaining on each postoperative day (%) and chest tube output is graded by showing different colors. **Supplementary Figure 2**: Blood coagulation test of the ABO group (n = 130) and the conventional TAR with FET group (n = 230). (**A**) D-dimer. (**B**) D-dimer ratio. (**C**) Fibrinogen degradation product. (**D**) Fibrinogen degradation product ratio. **Table S1**: Multivariable analysis of blood product transfusion*.

## Data Availability

The datasets used and/or analyzed during the current study are available from the corresponding author on reasonable request.

## Abbreviations

ABO: Aortic balloon occlusion
TAR: Total arch replacement
FET: Frozen elephant trunk
HCA: Hypothermic circulatory arrest
ALT: Alanine transaminase
AST: Aspartate transaminase
ICU: Intensive care unit
CA: Circulatory arrest
SD: Standard deviation
RBC: Red blood cell
AD: Aortic dissection
CPB: Cardiopulmonary bypass
FDP: Fibrinogen degradation product
NLR: Neutrophil/lymphocyte ratio
AKI: Acute kidney injury
CRRT: Continuous renal replacement therapy
