# Supplementary material for: Risk factor analysis of clinical outcomes of total aortic arch replacement and frozen elephant trunk with aortic balloon occlusion
Source: J Cardiothorac Surg. 2021 Sep 8;16:256. doi: 10.1186/s13019-021-01643-3 (PMC8425170; doi:10.1186/s13019-021-01643-3)
Supplement: Supplementary file 1 — Additional file 1. Supplementary Figure 1: Postoperative chest tube output of the ABO group (left column of each point, n = 130) and the conventional TAR with FET group (right column of each point, n = 230). Each bar represents the percentage of chest tube remaining on each postoperative day (%) and chest tube output is graded by showing different colors. Supplementary Figure 2: Blood coagulation test of the ABO group (n = 130) and the conventional TAR with FET group (n = 230). (A) D-dimer. (B) D-dimer ratio. (C) Fibrinogen degradation product. (D) Fibrinogen degradation product ratio. Table S1: Multivariable analysis of blood product transfusion*. [file 13019_2021_1643_MOESM1_ESM.docx]

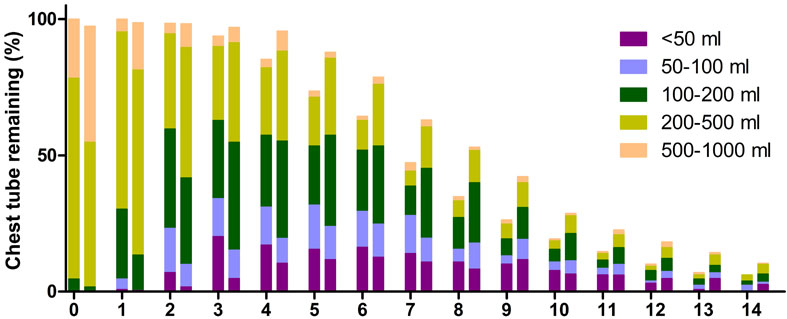


**Supplementary Figure 1**: Postoperative chest tube output of the ABO group (left column of each point, *n* = 130) and the conventional TAR with FET group (right column of each point, *n* = 230). Each bar represents the percentage of chest tube remaining on each postoperative day (%) and chest tube output is graded by showing different colors.

**
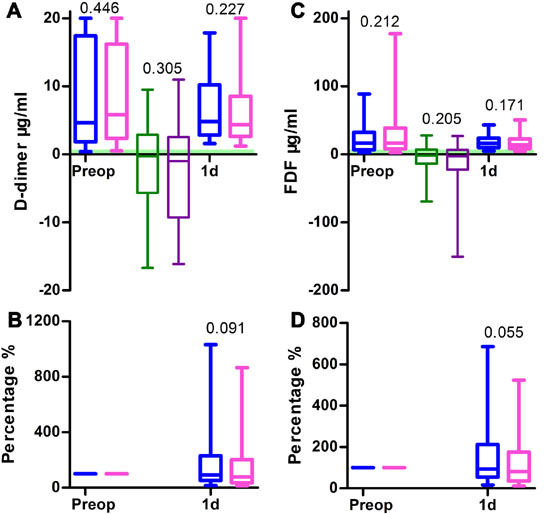
**

**Supplementary Figure 2**: Blood coagulation test of the ABO group (*n* = 130) and the conventional TAR with FET group (*n* = 230). (A) D-dimer. (B) D-dimer ratio. (C) Fibrinogen degradation product. (D) Fibrinogen degradation product ratio.

Table S1 Multivariable analysis of blood product transfusion*

| Risk factors (Hosmer-Lemeshow fitness†) | Odds ratio | 95% confidence interval | *P* |
| --- | --- | --- | --- |
| RBC during CPB (0.821) |  |  |  |
| Age (yrs) | 1.032 | 0.994-1.071 | 0.098 |
| Weight (kg) | 0.964 | 0.932-0.997 | 0.034 |
| Female | 3.685 | 1.338-10.147 | 0.012 |
| Cardiac surgery history | 6.108 | 1.341-27.818 | 0.019 |
| Preoperative hemoglobin (g/L) | 0.926 | 0.903-0.951 | <0.001 |
| Preoperative platelet count (10^9^/L) | 1.006 | 1.000-1.012 | 0.046 |
| Postoperative platelet count 0 (10^9^/L) | (0.993) | (0.983-1.003) | (0.158) |
| Preoperative leukocyte count (10^9^/L) | (1.033) | (0.918-1.163) | (0.590) |
| Preoperative percentage of neutrophils (%) | 0.965 | 0.926-1.006 | 0.091 |
| Preoperative FDP (μg/ml) | 1.006 | 1.000-1.012 | 0.045 |
| CPB time (min) | 1.008 | 1.003-1.013 | <0.001 |
| RBC during CPB ≥ 6 U (0.882) |  |  |  |
| Age (yrs) | 1.148 | 1.067-1.234 | <0.001 |
| Cardiac surgery history | 10.270 | 1.700-62.049 | 0.011 |
| Emergency operation | 8.175 | 1.072-62.345 | 0.043 |
| Preoperative hemoglobin (g/L) | 0.906 | 0.869-0.945 | <0.001 |
| Preoperative platelet count (10^9^/L) | 1.010 | 1.001-1.019 | 0.027 |
| Postoperative platelet count 0 (10^9^/L) | (0.995) | (0.979-1.012) | (0.550) |
| Preoperative leukocyte count (10^9^/L) | 1.232 | 0.996-1.524 | 0.054 |
| Preoperative percentage of neutrophils (%) | (0.987) | (0.915-1.065) | (0.737) |
| CPB time (min) | 1.016 | 1.008-1.023 | <0.001 |
| RBC post CPB (0.376) |  |  |  |
| Weight (kg) | 0.977 | 0.954-1.001 | 0.065 |
| Conventional group | 1.906 | 1.032-3.519 | 0.039 |
| Emergency operation | 2.725 | 1.091-6.806 | 0.032 |
| Preoperative hemoglobin (g/L) | 0.979 | 0.959-1.000 | 0.047 |
| Preoperative neutrophil count (10^9^/L) | 1.131 | 1.037-1.233 | 0.005 |
| CPB time (min) | 1.008 | 1.003-1.012 | <0.001 |
| RBC post CPB ≥ 6 U (0.826) |  |  |  |
| Height (cm) | 0.940 | 0.895-0.987 | 0.014 |
| Conventional group | 25.520 | 2.705-240.794 | 0.005 |
| Female | 0.095 | 0.012-0.739 | 0.024 |
| Preoperative AST (U/L) | 1.018 | 1.001-1.034 | 0.032 |
| Preoperative hemoglobin (g/L) | 0.947 | 0.908-0.987 | 0.009 |
| CPB time (min) | 1.008 | 1.002-1.014 | 0.008 |
| RBC total during operation (0.334) |  |  |  |
| Weight (kg) | 1.005 | 1.000-1.009 | 0.049 |
| Female | 4.322 | 1.600-11.672 | 0.004 |
| Cardiac surgery history | 7.703 | 1.348-44.016 | 0.022 |
| Preoperative hemoglobin (g/L) | 0.956 | 0.936-0.976 | <0.001 |
| CPB time (min) | 1.005 | 1.000-1.009 | 0.049 |
| RBC total during operation ≥ 6 U (0.496) |  |  |  |
| Age (yrs) | 1.088 | 1.038-1.141 | <0.001 |
| Conventional group | 3.554 | 1.394-9.057 | 0.008 |
| Cardiac surgery history | 8.760 | 2.064-37.177 | 0.003 |
| Emergency operation | 4.031 | 0.846-19.201 | 0.080 |
| Preoperative hemoglobin (g/L) | 0.913 | 0.883-0.943 | <0.001 |
| Preoperative platelet count (10^9^/L) | 1.018 | 1.010-1.026 | <0.001 |
| Postoperative platelet count 0 (10^9^/L) | 0.971 | 0.958-0.985 | <0.001 |
| Preoperative leukocyte count (10^9^/L) | 1.152 | 1.006-1.320 | 0.041 |
| Preoperative percentage of neutrophils (%) | (0.984) | (0.929-1.042) | (0.573) |
| CPB time (min) | 1.012 | 1.006-1.018 | <0.001 |
| RBC post-operation (0.149) |  |  |  |
| Emergency operation | 2.710 | 1.138-6.454 | 0.024 |
| CPB time (min) | 1.011 | 1.004-1.018 | 0.003 |
| Postoperative mechanical ventilation (h) | 1.016 | 1.005-1.028 | 0.006 |
| Postoperative hemoglobin 0† (g/L) | 0.925 | 0.899-0.951 | <0.001 |
| Postoperative platelet count 0 (10^9^/L) | 0.990 | 0.983-0.997 | 0.007 |
| Total chest tube drainage (per 100 ml) | 1.174 | 1.096-1.259 | <0.001 |
| RBC post-operation ≥ 6 U (0.748) |  |  |  |
| Weight (kg) | 0.948 | 0.909-0.989 | 0.014 |
| Height (cm) | 1.061 | 1.003-1.123 | 0.041 |
| Postoperative mechanical ventilation (h) | 1.023 | 1.013-1.034 | <0.001 |
| Postoperative hemoglobin 0 (g/L) | 0.961 | 0.932-0.992 | 0.013 |
| Postoperative platelet count 0 (10^9^/L) | 0.989 | 0.978-1.001 | 0.065 |
| Postoperative ALT1 (U/L) | 0.994 | 0.989-0.999 | 0.016 |
| Postoperative AST1 (U/L) | 1.004 | 1.000-1.007 | 0.027 |
| Postoperative Scr (μmol/L) | 1.011 | 1.003-1.019 | 0.006 |
| Platelet transfusion during operation (U) | 1.843 | 0.999-3.398 | 0.050 |
| Total chest tube drainage (per 100 ml) | 1.193 | 1.106-1.288 | <0.001 |
| RBC total (0.318) |  |  |  |
| Weight (kg) | 0.969 | 0.936-1.002 | 0.069 |
| Height (cm) | 0.938 | 0.876-1.004 | 0.066 |
| Female | 32.390 | 3.437-305.237 | 0.002 |
| Total abdominal aorta involvement | 3.097 | 1.354-7.084 | 0.007 |
| Preoperative hemoglobin (g/L) | 0.936 | 0.907-0.965 | <0.001 |
| Postoperative hemoglobin 0 (g/L) | 0.955 | 0.921-0.989 | 0.010 |
| CPB time (min) | 1.011 | 1.001-1.021 | 0.026 |
| Postoperative mechanical ventilation (h) | 1.020 | 1.003-1.038 | 0.020 |
| Postoperative Scr (μmol/L) | 1.008 | 0.999-1.016 | 0.068 |
| Total chest tube drainage (per 100 ml) | 1.167 | 1.067-1.277 | 0.001 |
| RBC total ≥ 6 U (0.431) |  |  |  |
| Age (yrs) | 1.033 | 0.997-1.071 | 0.074 |
| Weight (kg) | 0.959 | 0.928-0.911 | 0.012 |
| Conventional group | 2.134 | 1.107-4.110 | 0.024 |
| Preoperative hemoglobin (g/L) | 0.915 | 0.889-0.942 | <0.001 |
| Preoperative platelet count (10^9^/L) | 1.014 | 1.007-1.021 | <0.001 |
| Preoperative leukocyte count (10^9^/L) | 1.164 | 1.026-1.320 | 0.018 |
| Preoperative percentage of neutrophils (%) | 0.942 | 0.903-0.984 | 0.007 |
| CPB time (min) | 1.012 | 1.006-1.018 | <0.001 |
| Postoperative mechanical ventilation (h) | 1.015 | 1.005-1.024 | 0.002 |
| Postoperative platelet count 0 (10^9^/L) | 0.997 | 0.966-0.989 | <0.001 |
| Postoperative Scr (μmol/L) | 1.014 | 1.007-1.022 | <0.001 |
| Total chest tube drainage (per 100 ml) | 1.093 | 1.022-1.169 | 0.010 |
| Plasma during operation (0.803) |  |  |  |
| Total abdominal aorta involvement | 1.817 | 1.092-3.023 | 0.022 |
| CPB time (min) | 1.007 | 1.003-1.012 | 0.002 |
| Plasma during operation ≥ 600 ml (0.141) |  |  |  |
| Total abdominal aorta involvement | 2.066 | 1.145-3.727 | 0.016 |
| Preoperative leukocyte count (10^9^/L) | 1.091 | 1.022-1.187 | 0.046 |
| Preoperative percentage of neutrophils (%) | (0.976) | (0.943-1.010) | (0.170) |
| CPB time (min) | 1.006 | 1.002-1.010 | 0.002 |
| Plasma post-operation (0.204) |  |  |  |
| Postoperative mechanical ventilation (h) | 1.007 | 1.000-1.015 | 0.060 |
| Postoperative hemoglobin 0 (g/L) | 0.973 | 0.951-0.995 | 0.015 |
| Postoperative platelet count 0 (10^9^/L) | 1.006 | 1.000-1.012 | 0.056 |
| Total chest tube drainage (per 100 ml) | 1.123 | 1.064-1.186 | <0.001 |
| Plasma post-operation ≥ 600 ml (0.186) |  |  |  |
| Postoperative mechanical ventilation (h) | 1.006 | 1.000-1.012 | 0.066 |
| Postoperative Scr (μmol/L) | 1.006 | 1.000-1.012 | 0.069 |
| Total chest tube drainage (per 100 ml) | 1.164 | 1.088-1.244 | <0.001 |
| Plasma total (0.74) |  |  |  |
| Weight (kg) | 1.038 | 1.010-1.066 | 0.007 |
| CAD history | 3.515 | 1.226-10.079 | 0.019 |
| Postoperative mechanical ventilation (h) | 1.010 | 1.000-1.020 | 0.039 |
| Postoperative hemoglobin 0 (g/L) | 0.961 | 0.939-0.983 | 0.001 |
| Postoperative ALT1 (U/L) | 0.989 | 0.980-0.999 | 0.025 |
| Postoperative AST1 (U/L) | 1.008 | 1.000-1.016 | 0.048 |
| Total chest tube drainage (per 100 ml) | 1.116 | 1.049-1.187 | 0.001 |
| Plasma total ≥ 600 ml (0.775) |  |  |  |
| Weight (kg) | 1.035 | 1.009-1.062 | 0.008 |
| CPB time (min) | 1.008 | 1.002-1.013 | 0.007 |
| Postoperative mechanical ventilation (h) | 1.008 | 1.000-1.016 | 0.049 |
| Postoperative hemoglobin 0 (g/L) | 0.967 | 0.946-0.988 | 0.002 |
| Postoperative platelet count 0 (10^9^/L) | 0.988 | 0.980-0.996 | 0.004 |
| Total chest tube drainage (per 100 ml) | 1.098 | 1.039-1.160 | 0.001 |
| Platelet during operation (0.398) |  |  |  |
| Weight (kg) | 0.977 | 0.956-0.998 | 0.034 |
| CPB time (min) | 1.006 | 1.001-1.011 | 0.024 |
| Platelet post-operation (0.721) |  |  |  |
| CPB time (min) | 1.005 | 0.999-1.012 | 0.095 |
| Postoperative mechanical ventilation (h) | 1.009 | 1.000-1.018 | 0.047 |
| Preoperative Scr (μmol/L) | 1.006 | 1.001-1.012 | 0.030 |
| Platelet transfusion during operation (U) | 0.326 | 0.175-0.608 | <0.001 |
| Total chest tube drainage (per 100 ml) | 1.096 | 1.027-1.171 | 0.006 |
| Platelet post-operation ≥ 3 U (0.691) |  |  |  |
| Platelet count 0 (10^9^/L) | 0.954 | 0.919-0.990 | 0.013 |
| Postoperative leukocyte count 0 (10^9^/L) | (1.075) | (0.887-1.303) | (0.461) |
| Postoperative percentage of neutrophils 0 (%) | 1.304 | 1.006-1.689 | 0.045 |
| Postoperative Scr (μmol/L) | 1.012 | 1.000-1.025 | 0.049 |
| Platelet total (0.622) |  |  |  |
| Weight (kg) | 0.960 | 0.934-0.998 | 0.005 |
| CAD history | 0.288 | 0.094-0.888 | 0.030 |
| Preoperative platelet count (10^9^/L) | 0.990 | 0.985-0.996 | 0.001 |
| CPB time (min) | 1.012 | 1.005-1.020 | 0.002 |
| Postoperative platelet count 0 (10^9^/L) | 1.026 | 1.015-1.037 | <0.001 |
| Postoperative leukocyte count 0 (10^9^/L) | 0.917 | 0.843-0.997 | 0.042 |
| Postoperative percentage of neutrophils 0 (%) | (0.990) | (0.918-1.067) | (0.788) |
| Postoperative Scr (μmol/L) | 1.008 | 1.001-1.015 | 0.035 |
| Platelet total ≥ 3 U (0.903) |  |  |  |
| Female | 10.728 | 1.726-66.683 | 0.011 |
| Postoperative platelet count 0 (10^9^/L) | 0.971 | 0.953-0.989 | 0.002 |
| Postoperative FDP (μg/mL) | 1.035 | 1.003-1.069 | 0.033 |
| Total chest tube drainage (per 100 mL) | 1.184 | 1.071-1.308 | 0.001 |

*Blood product transfusion was performed with binary logistic regression for if transfusion is required (>0 package) or large transfusion is required (≥3 packages).

†Each multivariable analysis of risk factors can be accepted if the *P* value of Hosmer-Lemeshow fitness was more than 0.05.

‡All the postoperative blood routine 0 stands for the immediate reexamination time point when the operation ended and transferred to ICU.

§All the postoperative blood test 1 stands for the reexamination time point of postoperative day 1.

ALT = alanine transaminase; AST = aspartate transaminase; CAD = coronary artery disease; CPB = cardiopulmonary bypass; FDP = fibrinogen degradation product; RBC = red blood cell count; Scr = serum creatinine.
